# Supplementary material for: Signal Deconvolution and Generative Topographic Mapping Regression for Solid-State NMR of Multi-Component Materials
Source: Int J Mol Sci. 2021 Jan 22;22(3):1086. doi: 10.3390/ijms22031086 (PMC7865946; doi:10.3390/ijms22031086)
Supplement: Supplementary file 1 [file ijms-22-01086-s001.pdf]

International Journal of *Molecular Sciences*

Supplementary materials for

Signal Deconvolution and Generative Topographic Mapping Regression for Solid-state NMR of Multi-component Materials

Shunji Yamada [1,2], Eisuke Chikayama [2,3] and Jun Kikuchi [1,2,4,*]

1 Graduate School of Bioagricultural Sciences, Nagoya University, Furo-cho, Chikusa-ku, Nagoya 464-8601, Japan; shunji.yamada@riken.jp (S.Y.)
2 Environmental Metabolic Analysis Research Team, RIKEN Center for Sustainable Resource Science, 1-7-22 Suehiro-cho, Tsurumi-ku, Yokohama 230-0045, Japan; chikaya@nuis.ac.jp (E.C.)
3 Department of Information Systems, Niigata University of International and Information Studies, 3-1-1 Mizukino, Nishi-ku, Niigata 950-2292, Japan
4 Graduate School of Medical Life Science, Yokohama City University, 1-7-29 Suehiro-cho, Tsurumi-ku, Yokohama 230-0045, Japan
* Correspondence: jun.kikuchi@riken.jp (J.K.); +81-45-508-7220 (J.K.)

In this document, we provide the supplementary information.

Supplementary figures

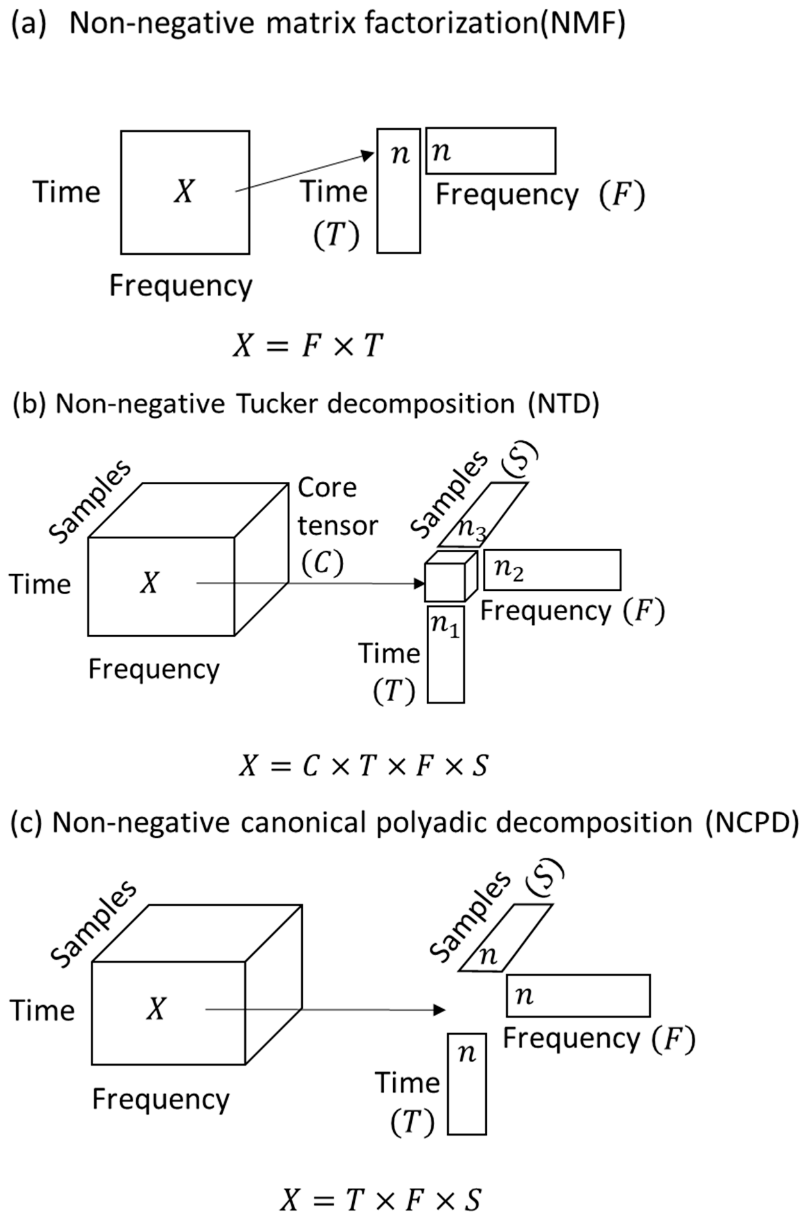

Figure S1. Algorithms of non-negative tensor/matrix factorization (NTF, NMF). (a) Non-negative matrix factorization (NMF). (b) Non-negative Tucker decomposition (NTD). (c) Non-negative canonical polyadic decomposition (NCPD). In the case of two-dimensional datasets such as a matrix with time and frequency axes, the FID is separated into each component based on factors of time and frequency by matrix factorization. For analysis of the three-dimensional dataset of multiple samples and parameters, tensor methods such as NTD and NCPD can be used.

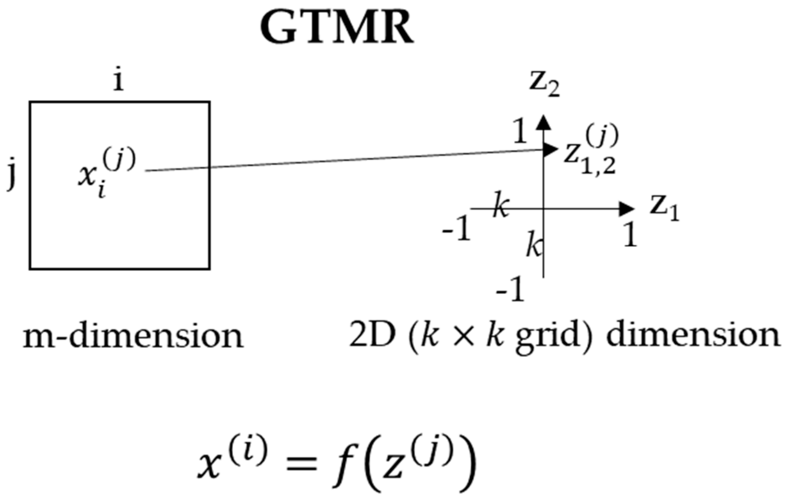

Figure S2. Algorithm of generative topographic mapping regression (GTMR). Using the GTMR, multi-dimensional and multi-component data can be mapped into the reduced dimensional space.

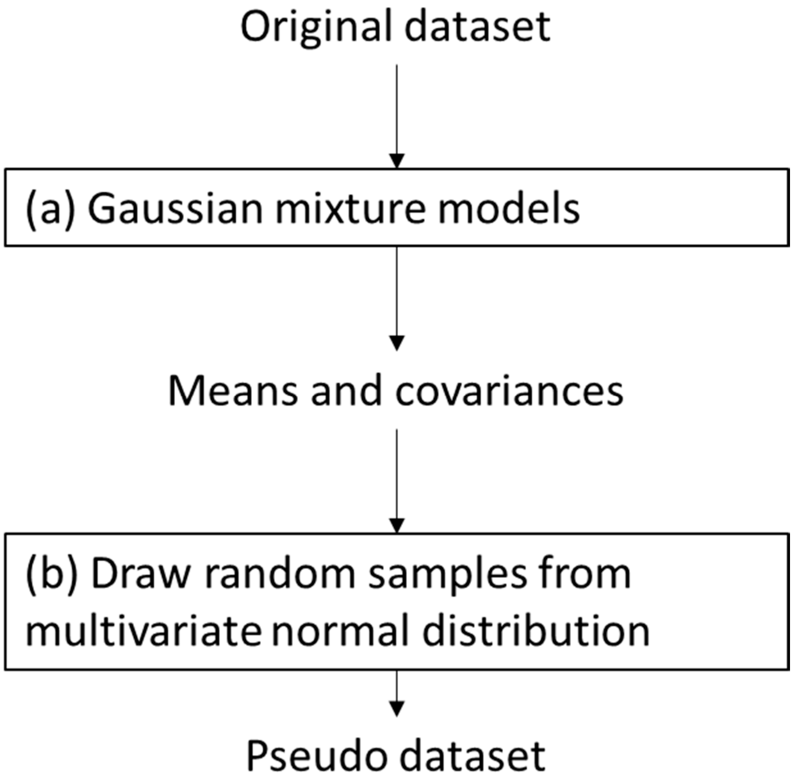

Figure S3. Algorithm of generating data using Gaussian mixture models (GMM). (a) GMM estimates the distribution of the dataset. (b) Draw random samples based on distribution estimated by GMM.

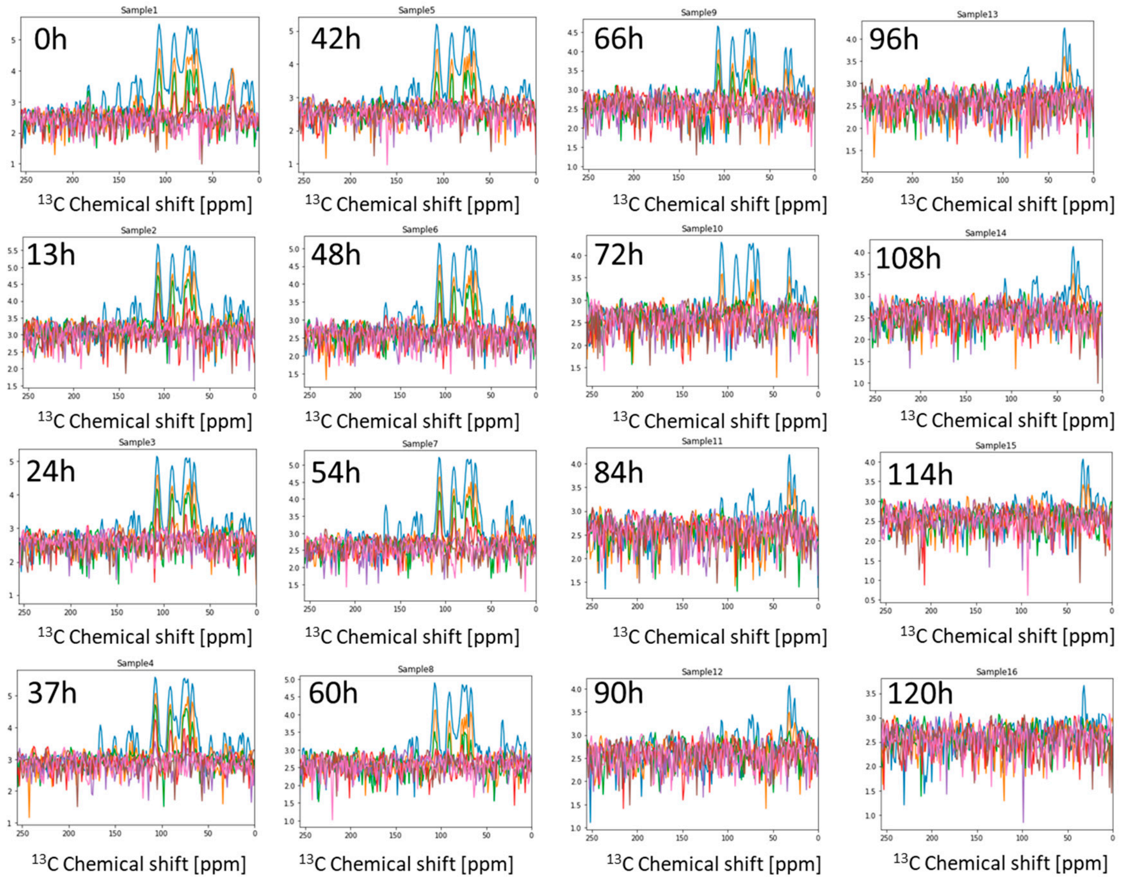

Figure S4. Short-time Fourier transform processed NMR (STFT-NMR) signals in ^{13}C CP-MAS of the cellulose degradation process. These figures show STFT processed NMR data for each time of the cellulose degradation process.

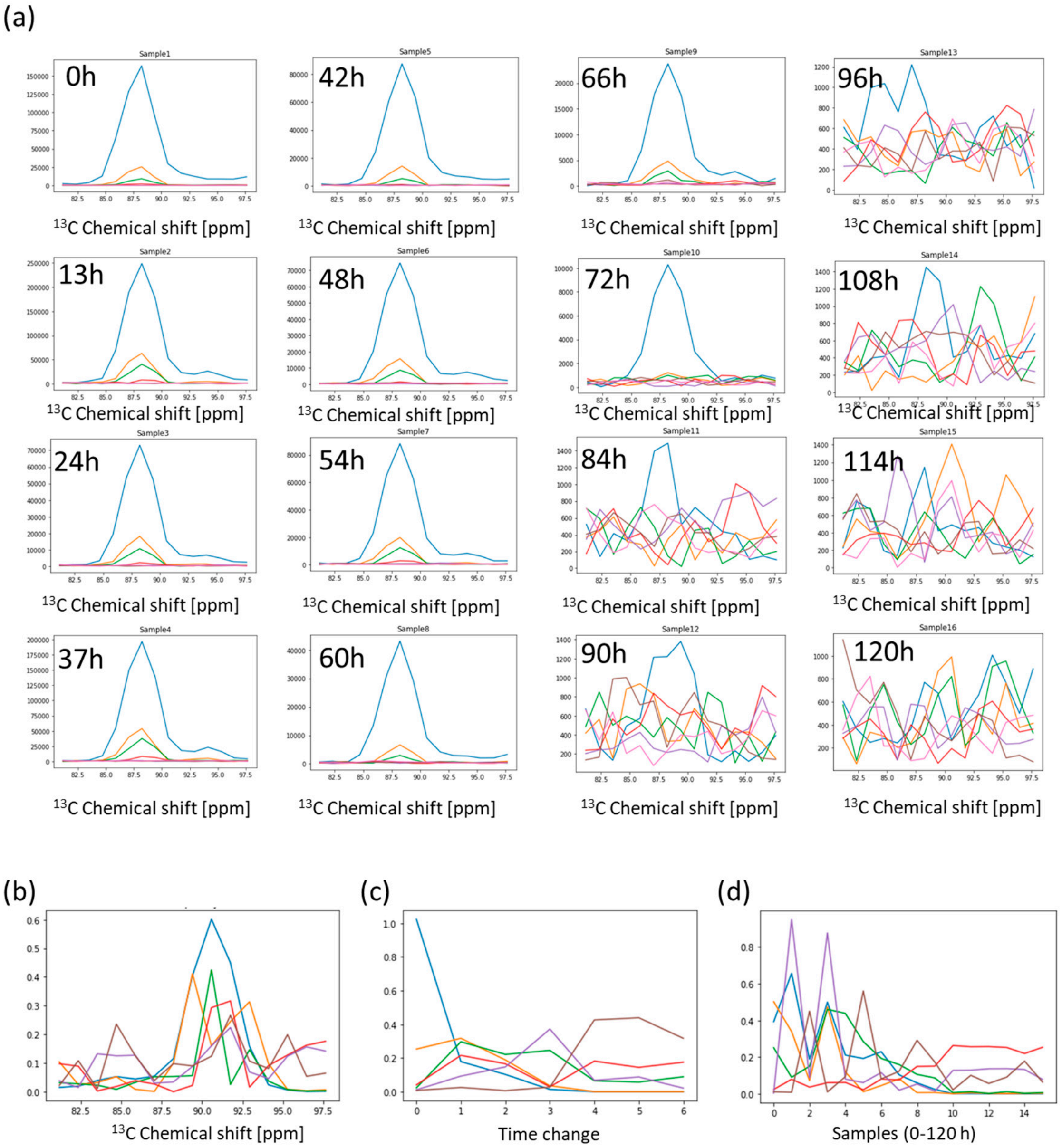

Figure S5. Signal deconvolution of cellulose C4 region using non-negative Tucker decomposition (NTD) in ^{13}C CP-MAS of cellulose degradation process. (a) These figures show cellulose C4 region STFT processed NMR data for each time of the cellulose degradation process. The figures (b-d) show spectral patterns (b), time change of separated components (5c), and composition of separated components (d) as results of separating the spectrum of cellulose C4 region into six components.

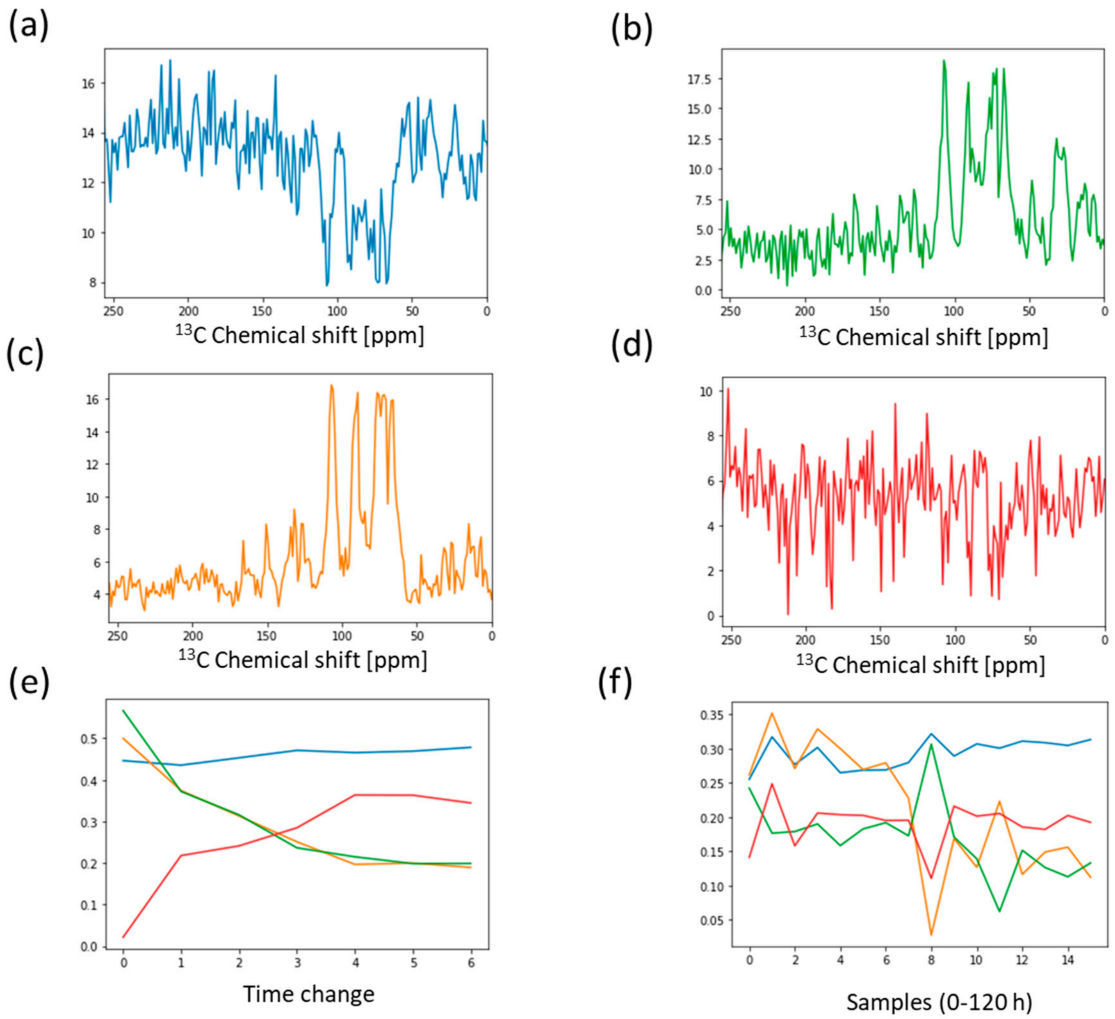

Figure S6. Signal deconvolution using non-negative canonical polyadic decomposition (NCPD) in ^{13}C CP-MAS of the cellulose degradation process. These figures show spectral patterns (a-d), time change of separated components (e), and composition of separated components (f) as results of separating the spectrum of cellulose using NCPD.

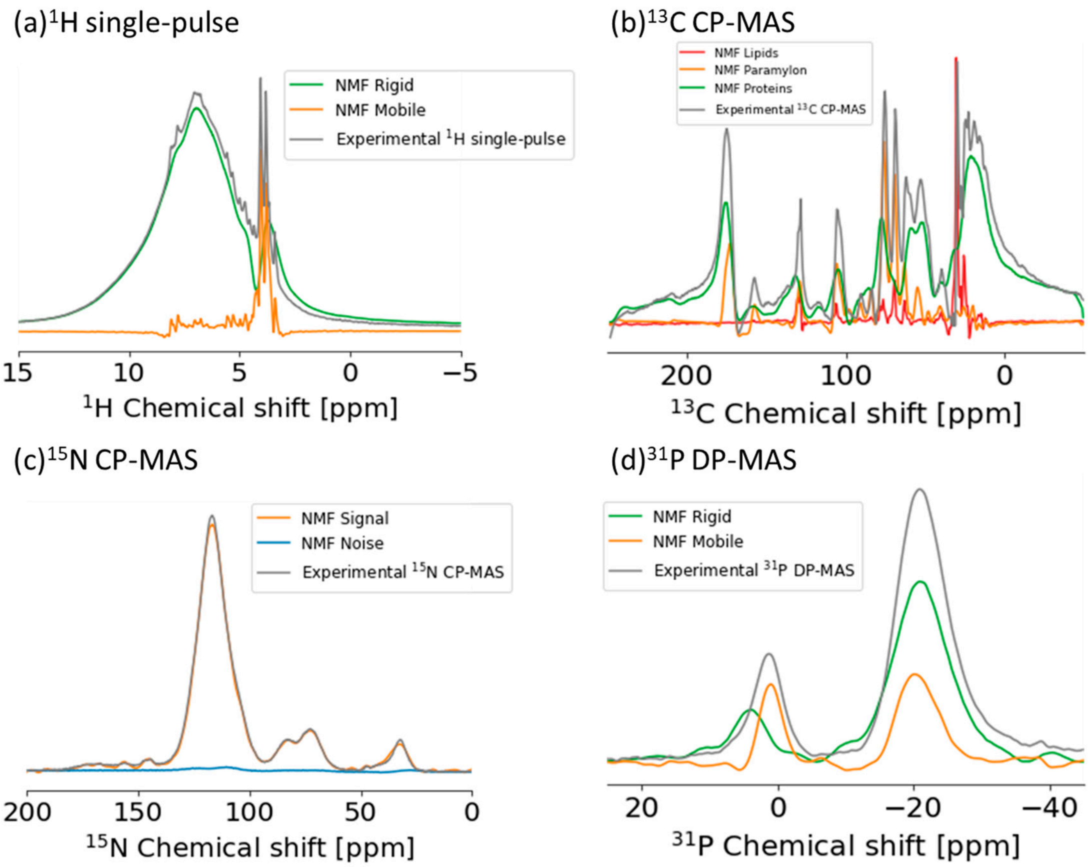

Figure S7. Signal deconvolution using MF to various NMR spectra in *E. graclis* samples. These figures show results of the signal deconvolution method using NMF for ^{1}H (a), ^{13}C (b), ^{15}N (c) and ^{31}P (d) spectra of microalgae such as *E. gracilis* in a multi-component system.

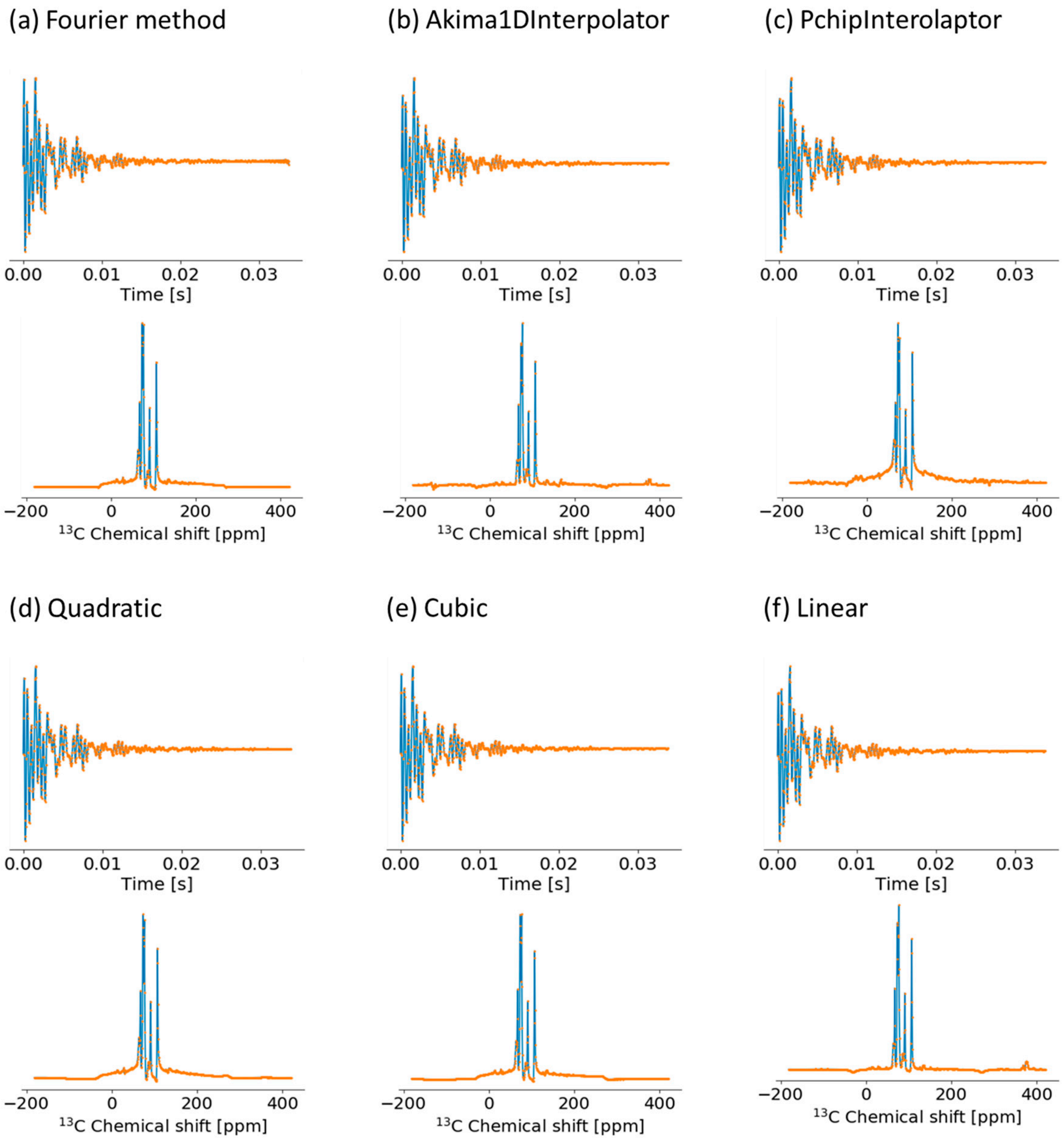

Figure S8. Application of interpolation methods for signal deconvolution of NMR data with insufficient data points. These figures show results of the resampling method using Fourier method (a) and other interpolation methods such as Akima, PCHIP (Piecewise Cubic Hermite Interpolating Polynomial), quadratic, cubic and linear (b-f).

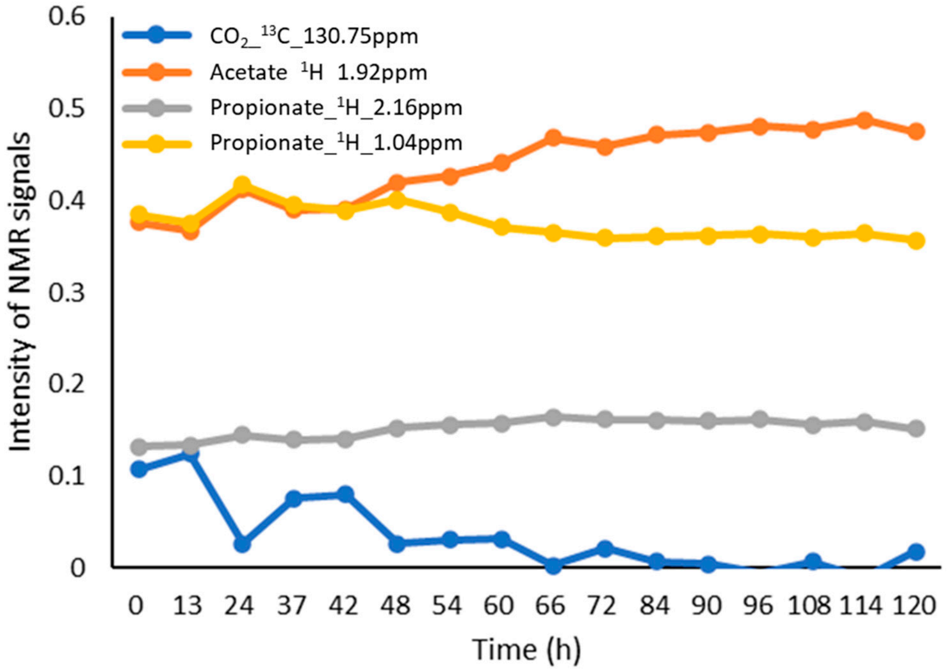

Figure S9. Summary of NMR signals for prediction in the cellulose degradation process. This figure shows the cellulose degradation process such as CO_2 (^{13}C chemical shift is 130.75 ppm), acetate (^{1}H chemical shift is 1.92 ppm), propionate (^{1}H chemical shift is 2.16 and 1.04 ppm) was captured by solution NMR.

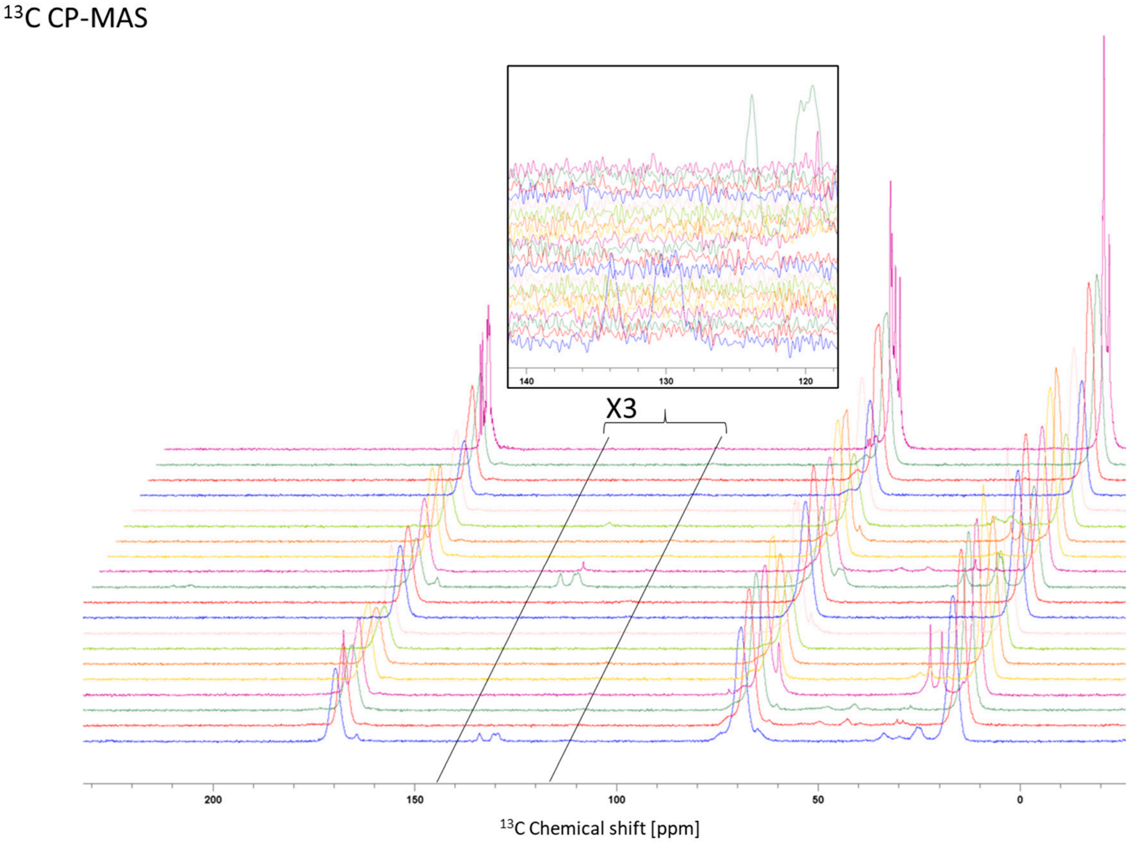

Figure S10. Summary of NMR data for prediction in polylactic acid (PLA). This figure shows ^{13}C CP-MAS spectra of 22 plastics.

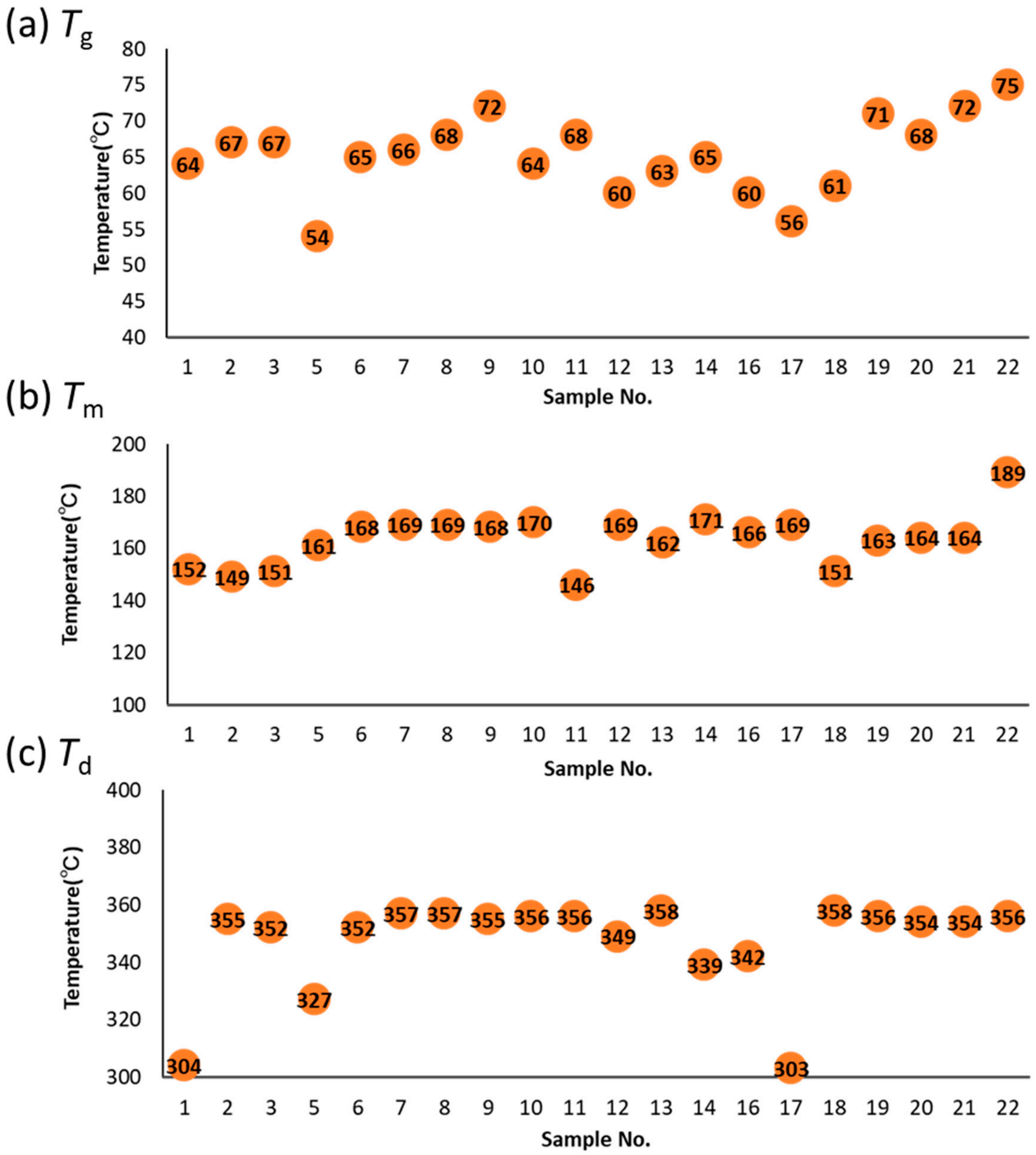

Figure S11. Summary of thermal analysis data for prediction in PLA. These figures show thermal analysis data of T_g (a), T_m (b), T_d (c) in 22 plastics.

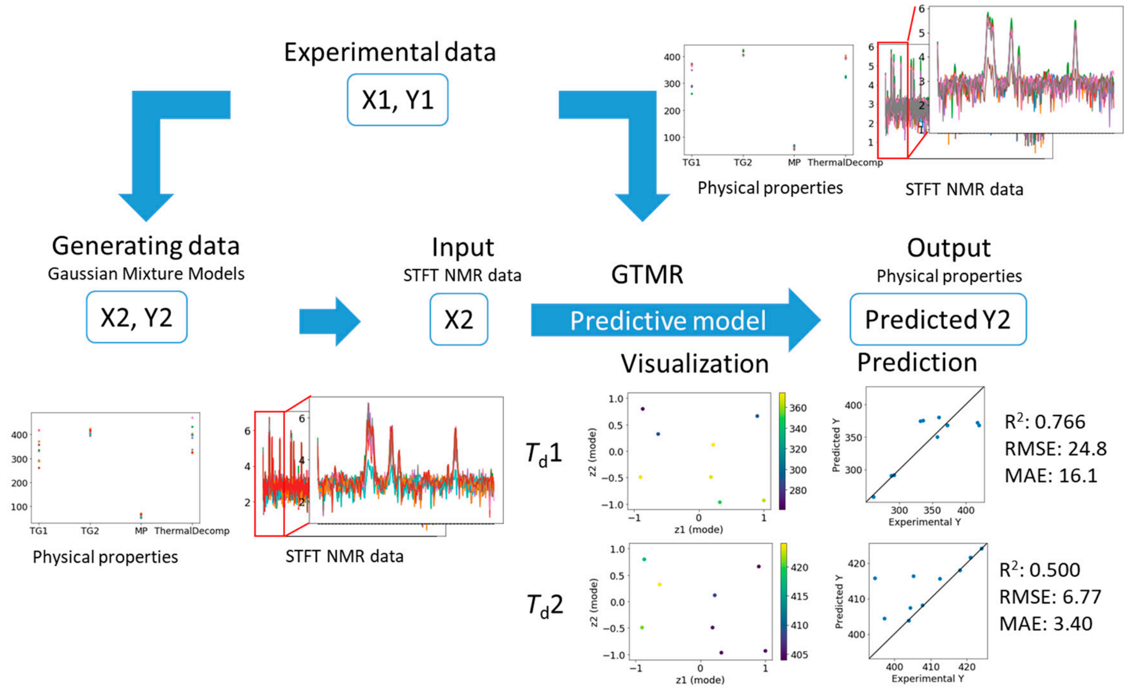

Figure S12. Prediction to thermal properties from NMR signals generated Gaussian mixture models (GMM) in poly-ε-caprolactone. This figure shows a scheme and result of predicting the thermal properties such as the degradation temperature (T_d)from pseudo ^{13}C CP-MAS spectra using GMM.
